# Physician Perspectives of Overdiagnosis and Overtreatment of Low-Risk Papillary Thyroid Cancer in the US

**DOI:** 10.1001/jamanetworkopen.2022.8722

**Published:** 2022-04-25

**Authors:** Priya H. Dedhia, Megan C. Saucke, Kristin L. Long, Gerard M. Doherty, Susan C. Pitt

**Affiliations:** 1 Division of Surgical Oncology, Department of Surgery, The Ohio State University Comprehensive Cancer Center, The Ohio State University Wexner Medical Center, Columbus; 2 Wisconsin Surgical Outcomes Research Program, Department of Surgery, University of Wisconsin School of Medicine and Public Health, Madison; 3 Division of Endocrine Surgery, Department of Surgery, University of Wisconsin School of Medicine and Public Health, Madison; 4 Brigham and Women’s Hospital, Brigham Health, Dana-Farber Cancer Institute, Harvard Medical School, Boston, Massachusetts; 5 Department of Surgery, University of Michigan, Ann Arbor

## Abstract

This survey study assesses physicians’ recommendations regarding overdiagnosis and overtreatment of thyroid nodules and low-risk papillary thyroid cancer.

## Introduction

Overdiagnosis and overtreatment of low-risk thyroid cancer are important problems.^1^ American Thyroid Association (ATA) guidelines indicate that thyroid nodules less than 1 cm should not be biopsied, nodules 1 cm to 1.5 cm should be biopsied only when features concerning for a malignant tumor exist, and papillary thyroid cancer (PTC) nodules 1 cm or less should be managed with active surveillance or lobectomy.^2^ Biopsy and treatment with total thyroidectomy or radioactive iodine (RAI) outside these recommendations have been associated with overdiagnosis and overtreatment, respectively. Because physician-level factors associated with overdiagnosis and overtreatment of thyroid cancer are poorly understood, we conducted a national survey examining physicians’ recommendations for thyroid nodules and low-risk PTC.

## Methods

In this survey study, surveys were mailed to 1500 endocrinologists, general surgeons, and otolaryngologists (500 each) randomly selected from the American Medical Association Physician Masterfile in August 2018. Survey development and administration were previously described (eAppendix in the Supplement).^3^ Respondents who were actively practicing, had treated thyroid cancer since 2015 (ATA guidelines publication date), and had responded to questions about overdiagnosis (biopsy outside ATA guideline for nodules <1 cm with suspicious features or nodules <1.5 cm without suspicious features) and overtreatment (total or completion thyroidectomy, central neck dissection, and/or RAI for 1 low-risk PTC <1 cm) were included. Responses were calculated using the AAPOR guideline (response rate 2), in which all nonresponders are considered eligible. Questions considered demographics, guideline use, and management of thyroid nodules and cancer. Statistical analysis included Fisher exact, Wilcoxon rank sum, and *t* tests as appropriate (2-sided *P* < .05 was significant). The University of Wisconsin institutional review board deemed the study exempt because use of surveys involved minimal risk to participants. Survey participation was voluntary, and survey completion indicated consent; responses were deidentified. R statistical software, version x.y.z., was used. Data were analyzed from December 2019 to August 2020.

## Results

Of 1500 individuals sent surveys, 487 (32.5%) responded; 439 were eligible for analysis (Table 1). Respondents’ demographics were similar to those of Association of American Medical Colleges active physicians in each specialty. Nonrespondents were demographically similar to respondents but more likely to be female.^3^

**Table 1.** Survey Respondent Characteristics by Diagnostic Preferences

Characteristic	Respondents^a^			*P* value
	Overdiagnosis (n = 280)^b^	Appropriate care (n = 159)	Total (N = 439)	
Age, mean (SD), y	53.0 (9.1)	54.4 (8.9)	53.5 (9.1)	.12
Gender				
Female	56 (20.4)	36 (23.2)	92 (21.5)	.64
Male	217 (79.2)	118 (76.1)	335 (78.1)	
Other	1 (0.4)	1 (0.6)	2 (0.5)	
Race and ethnicity^c^				
American Indian/Alaskan Native	0	0	0	.27
Asian	40 (14.7)	18 (12.2)	58 (13.8)	
Black	4 (1.5)	2 (1.4)	6 (1.4)	
Hispanic/Latino	9 (3.3)	5 (3.4)	14 (3.3)	
White	211 (77.3)	111 (75.0)	322 (76.5)	
Other^d^	8 (2.9)	8 (5.4)	16 (3.8)	
>1 Race or ethnicity	1 (0.4)	4 (2.7)	5 (1.2)	
Time in practice, mean (SD), y	20.1 (9.1)	21.5 (9.0)	20.6 (9.1)	.13
Time since training, y				
0 to <5	12 (4.4)	6 (3.8)	18 (4.2)	.16
5 to <10	45 (16.4)	21 (13.5)	66 (15.3)	
10 to <20	90 (32.8)	39 (25.0)	129 (27.9)	
≥20	127 (46.4)	90 (57.7)	217 (50.4)	
Specialty				
Endocrinology	93 (33.2)	46 (28.9)	139 (31.7)	.26
General surgery	65 (23.2)	48 (30.1)	113 (25.7)	
Otolaryngology	122 (43.6)	65 (40.9)	187 (42.6)	
Location				
Northeast	53 (19.8)	36 (23.8)	89 (21.2)	.21
Midwest	66 (24.6)	47 (31.1)	113 (27.0)	
South	101 (37.7)	48 (31.8)	149 (35.6)	
West	48 (17.9)	20 (13.3)	68 (16.2)	
Practice setting				
Academic tertiary hospital	38 (13.7)	24 (15.4)	62 (14.3)	.46
Academic-affiliated hospital	32 (11.6)	11 (7.1)	43 (9.9)	
Community	57 (20.6)	36 (23.1)	93 (21.5)	
Private practice	144 (52.0)	79 (50.6)	223 (51.5)	
Other	6 (2.2)	6 (3.8)	12 (2.8)	
Access to tumor board to discuss patient management				
Yes	202 (72.7)	109 (69.9)	311 (71.7)	.17
No	71 (25.5)	47 (30.1)	118 (27.2)	
Not applicable	5 (1.8)	0	5 (1.2)	
Uses 2015 ATA guidelines				
Yes	223 (86.4)	119 (82.1)	342 (84.9)	.25
No	35 (13.6)	26 (17.9)	61 (15.1)	
Primarily responsible for deciding whether a thyroid nodule needs FNA				
Myself	224 (80.0)	128 (80.5)	352 (80.2)	>.99
Other	56 (20.0)	31 (19.5)	87 (19.8)	
Primarily performs FNA for patients with thyroid nodules				
Myself	100 (35.7)	62 (39.0)	352 (36.9)	.54
Other	180 (64.3)	97 (61.0)	87 (63.1)	

Abbreviations: ATA, American Thyroid Association; FNA, fine-needle aspiration.

^a^ Data are reported as number (percentage) of respondents unless otherwise indicated. Totals in each category may not sum to column total because some respondents omitted responses to survey questions in that category.

^b^ Defined as recommending fine-needle biopsy for patients outside the 2015 ATA guidelines for nodules less than 1 cm with features concerning for papillary thyroid cancer or nodules less than 1.5 cm without concerning features.

^c^ Race and ethnicity were self-reported.

^d^ Other includes Arab American, Indian American, Indian subcontinent, multiracial (identifying as 50% Asian and 50% White), and Pakistani.

Overdiagnosis was recommended by 280 respondents (64.0%). No significant demographic, specialty, or regional differences existed between respondents recommending overdiagnosis vs appropriate care (Table 1). Regarding low-risk PTC treatment, 178 (42.5%), 265 (63.3%), and 263 (63.7%) respondents believed total thyroidectomy, total thyroidectomy with central neck dissection, and RAI, respectively, were overused (Table 2). Beliefs regarding overuse did not vary significantly based on propensity for overdiagnosis.

**Table 2.** Respondents’ Beliefs About, Recommendations for, and Factors Influencing Treatment for Low-Risk Thyroid Cancer

	Respondents, No. (%)			*P* value
	Overdiagnosis (n = 280)^a^	Appropriate care (n = 159)^a^	Total (N = 439)^a^	
Beliefs				
Total thyroidectomy				
Overused	104 (39.0)	74 (48.7)	178 (42.5)	.15
Appropriately used	156 (58.4)	75 (49.3)	231 (55.1)	
Underused	7 (2.6)	3 (2.0)	10 (2.4)	
Total thyroidectomy with central neck dissection				
Overused	159 (60.0)	106 (68.8)	265 (63.3)	.18
Appropriately used	89 (33.6)	42 (27.3)	131 (31.3)	
Underused	17 (6.4)	6 (3.9)	23 (5.5)	
Radioactive iodine				
Overused	166 (63.4)	97 (64.2)	263 (63.7)	>.99
Appropriately used	91 (34.7)	52 (34.4)	143 (34.6)	
Underused	5 (1.9)	2 (1.3)	7 (1.7)	
Recommends overtreatment^b^				
Yes	119 (43.0)	56 (35.4)	175 (40.2)	.004
No	158 (57.0)	102 (64.6)	260 (59.8)	
Factors influencing decision to recommend a particular treatment				
Risk of complications				
A great deal/quite a bit	127 (46.2)	68 (43.9)	195 (45.4)	.09
Some	104 (37.8)	49 (31.6)	153 (35.6)	
A little/none	44 (16.0)	38 (24.5)	82 (19.1)	
Peace of mind from more extensive surgery				
A great deal/quite a bit	76 (27.8)	33 (21.2)	109 (25.4)	.03
Some	97 (35.5)	45 (28.8)	142 (33.1)	
A little/none	100 (36.6)	78 (50.0)	178 (41.5)	
Concern about doing less-extensive surgery				
A great deal/quite a bit	77 (28.3)	28 (18.2)	105 (24.7)	.06
Some	120 (44.1)	75 (48.7)	195 (45.8)	
A little/none	75 (27.6)	51 (33.1)	105 (24.7)	
Need for life-long thyroid hormone replacement				
A great deal/quite a bit	65 (24.0)	30 (19.2)	95 (22.3)	.51
Some	98 (36.2)	61 (39.1)	159 (37.2)	
A little/none	108 (39.9)	65 (41.7)	173 (40.5)	
Risk of cancer recurrence				
A great deal/quite a bit	165 (60.0)	97 (62.2)	262 (60.8)	.16
Some	79 (28.7)	34 (21.8)	113 (26.2)	
A little/none	31 (11.3)	25 (16.0)	56 (13.0)	
Ease of follow-up				
A great deal/quite a bit	121 (45.0)	55 (35.5)	176 (41.5)	.14
Some	84 (31.2)	60 (38.7)	144 (34.0)	
A little/none	64 (23.8)	40 (25.8)	104 (24.5)	
Patient reliability to follow-up				
A great deal/quite a bit	134 (50.0)	75 (48.1)	209 (49.3)	.87
Some	86 (32.1)	50 (32.1)	136 (32.1)	
A little/none	108 (39.9)	65 (41.7)	173 (40.5)	
Ability to follow thyroglobulin when recommending a completion thyroidectomy				
A great deal/quite a bit	134 (63.8)	48 (45.3)	182 (57.6)	.002
Some	76 (36.2)	58 (54.7)	134 (42.4)	
A little/none	0	0	0	

^a^ Totals in each category may not sum to column total because some respondents omitted responses to survey questions in that category.

^b^ Defined as recommending total thyroidectomy with or without central neck dissection for a 0.8-cm papillary thyroid cancer and/or completion thyroidectomy or radioactive iodine for patient with a solitary low-risk papillary thyroid cancer.

Overtreatment was recommended by 175 respondents (40.2%) (Table 2). Respondents who favored overdiagnosis were more likely to recommend overtreatment of low-risk PTC surgically or with RAI (119 [43.0%] vs 56 [35.4%]; *P* = .004) and to be influenced by peace of mind from more extensive surgery (173 of 273 [63.4%] vs 78 of 156 [50.0%]; *P* = .03).

## Discussion

A total of 64.0% of respondents recommended overdiagnosis of small thyroid nodules. Physicians favoring overdiagnosis were more likely to recommend overtreatment of low-risk PTC with surgery or RAI, although not consistently across scenarios. Physicians favoring overdiagnosis also indicated that peace of mind from more extensive surgery influenced their recommendations. Need for diagnostic certainty and fear of missing a diagnosis have been associated with overdiagnosis and overtreatment in other contexts.^4,5^ Although our study may be limited by nonresponse bias, we found no differences between early and late respondents and between respondents and nonrespondents. Our study did not include primary care physicians, who may also influence the decision to pursue thyroid nodule biopsy. Successful implementation of guidelines, which can take 17 years, requires dissemination, education, and decision guides.^6^ Future research may provide insight into improving adherence to ATA guidelines.

Reducing overdiagnosis may be an important strategy for reducing overtreatment. However, because not all physicians who favored overdiagnosis recommended overtreating low-risk PTC, additional strategies are necessary to reduce overtreatment.

## Notes

Supplement. **eAppendix.** National Study of Providers’ Practices for Thyroid Cancer
