# Supplementary material for: Physician Perspectives of Overdiagnosis and Overtreatment of Low-Risk Papillary Thyroid Cancer in the US
Source: JAMA Netw Open. 2022 Apr 25;5(4):e228722. doi: 10.1001/jamanetworkopen.2022.8722 (PMC9039765; doi:10.1001/jamanetworkopen.2022.8722)
Supplement: Supplement. — eAppendix. National Study of Providers’ Practices for Thyroid Cancer [file jamanetwopen-e228722-s001.pdf]

Supplemental Online Content

Dedhia PH, Saucke MC, Long KL, Doherty GM, Pitt SC. Physician perspectives of overdiagnosis and overtreatment of low-risk papillary thyroid cancer in the US. *JAMA Netw Open*. 2022;5(4):e228722. doi:10.1001/jamanetworkopen.2022.8722

eAppendix. National Study of Providers' Practices for Thyroid Cancer

This supplemental material has been provided by the authors to give readers additional information about their work.

© 2022 Dedhia PH et al. *JAMA Network Open.*

Wisconsin Surgical Outcomes
Research Program
Department of Surgery
UNIVERSITY OF WISCONSIN
SCHOOL OF MEDICINE AND PUBLIC HEALTH

National Study of Providers' Practices for Thyroid Cancer

You can skip any questions that you do not want to answer. Please also feel free to write any additional comments you may have. For the purposes of this survey, *active surveillance* refers to performing serial physical exams, ultrasounds, and/or laboratory tests on a patient with biopsy-proven thyroid cancer, but who is not undergoing surgery at this time, also called "watchful waiting."

1. The first several questions are about your practice.

Which of the following best describes your specialty?

○ Endocrinology
○ General surgery
○ Otolaryngology
○ Other → Please tell us: ____

2. Does your current practice include a focus on thyroid disease?

○ Yes
○ No

3. Since December 2015, have you cared for any patients with thyroid nodules or thyroid cancer?

○ Yes
○ No → **Go to question 29**

4. In the last 12 months, about how many new or established patients with thyroid cancer have you cared for?

○ 5 or fewer
○ 6 to 10
○ 11 to 25
○ 26 to 50
○ More than 50

If you are a surgeon → ***Go to question 5***

If you are not a surgeon → ***Go to question 7***

5. In a 12 month period, about how many thyroid surgeries do you typically perform?

© 2022 Dedhia PH et al. *JAMA Network Open.*

[ ] thyroid surgeries

6. Who <u>primarily</u> performs FNA on your patients with thyroid nodules?

○ Myself
○ Surgeon
○ Endocrinologist
○ Radiologist
○ Other → Please tell us: [ ]

7. At what size do you recommend FNA of a thyroid nodule <u>with</u> features concerning for papillary thyroid cancer, such as microcalcifications, irregular borders, and increased vascularity?

[ ] Centimeters (1 cm = 10 mm) or larger

8. At what size do you recommend FNA of a thyroid nodule <u>without</u> any concerning features?

[ ] Centimeters (1 cm = 10 mm) or larger

9. The next questions are about management of <u>low-risk</u> thyroid cancer and factors that may influence your recommendations.

For this question, consider a healthy <u>45-year old</u> female patient with a <u>biopsy-proven,</u> <u>solitary</u> papillary thyroid cancer. In addition, assume she has:

- **<u>No</u> contralateral nodules**
- **<u>No</u> lymphadenopathy**
- **<u>No</u> extrathyroidal extension**
- **<u>No</u> family history of thyroid cancer or history of head and neck radiation**

For each of the following scenarios, please indicate which management option you would recommend if you could recommend <u>only one</u> approach.

Would you recommend total thyroidectomy with central neck dissection, total thyroidectomy, thyroid lobectomy, or active surveillance for a cancer that is…

	Total thyroidectomy with central neck dissection	Total thyroidectomy	Thyroid lobectomy	Active surveillance
**a.** …0.8 cm?	○	○	○	○
**b.** …0.8 cm and patient prefers total thyroidectomy?	○	○	○	○

© 2022 Dedhia PH et al. *JAMA Network Open.*

10. Think about your decision to recommend a particular treatment for patients with a solitary, low-risk thyroid cancer.

How much influence does each of the following factors have on your decision?

	None	A little	Some	Quite a bit	A great deal
**a.** Clinical characteristics, such as size of the cancer or patient age	○	○	○	○	○
**b.** Risks of complications	○	○	○	○	○
**c.** Risk of cancer recurrence	○	○	○	○	○
**d.** Ease of follow-up	○	○	○	○	○
**e.** Patient reliability to follow-up	○	○	○	○	○
**f.** Peace of mind from more extensive surgery	○	○	○	○	○
**g.** Need for life-long thyroid hormone replacement	○	○	○	○	○
**h.** Recommendation of referring provider	○	○	○	○	○
**i.** Patient preference	○	○	○	○	○
**j.** Concern about doing less extensive surgery	○	○	○	○	○

11. Think about patients who initially underwent a thyroid lobectomy for an indeterminate nodule and were found to have a low-risk papillary thyroid cancer on final pathology.

For whom do you recommend a completion thyroidectomy?

○ For all patients
○ For most patients
○ For select patients
○ Only for patients who have an incidental positive lymph node on final pathology
○ For no patients

12. Think about your decision whether to recommend a completion thyroidectomy for a patient with a low-risk papillary thyroid cancer.

How much influence does each of the following factors have on your decision?

	None	A little	Some	Quite a bit	A great deal
**a.** Clinical characteristics, such as size of the cancer or multifocality	○	○	○	○	○
**b.** Ability to perform radioactive iodine	○	○	○	○	○
**c.** Ability to follow thyroglobulin	○	○	○	○	○
**d.** Need for life-long thyroid hormone replacement	○	○	○	○	○
**e.** Patient preference for a completion	○	○	○	○	○
**f.** Patient preference for no further surgery	○	○	○	○	○
**g.** Concern about the remaining thyroid tissue	○	○	○	○	○

© 2022 Dedhia PH et al. *JAMA Network Open.*

13. Think about patients with low-risk papillary thyroid cancer.

For whom do you recommend central neck dissection?

- ◯ For all patients
- ◯ For most patients
- ◯ For select patients
- ◯ Only for patients who have clinically positive lymph nodes on ultrasound or discovered intraoperatively
- ◯ For no patients

14. Think about patients with low-risk papillary thyroid cancer. For whom do you recommend RAI?

- ◯ For all patients
- ◯ For most patients
- ◯ For select patients
- ◯ Only for patients who have positive lymph nodes on final pathology
- ◯ For no patients

15. Think about your decision whether to recommend RAI for a patient with low-risk thyroid cancer.

How much influence does each of the following factors have on your decision?

	None	A little	Some	Quite a bit	A great deal
**a.** Clinical characteristics, such as size of the cancer or patient age	◯	◯	◯	◯	◯
**b.** Risk of side effects	◯	◯	◯	◯	◯
**c.** Risk of cancer recurrence	◯	◯	◯	◯	◯
**d.** Patient reliability to follow-up	◯	◯	◯	◯	◯
**e.** Peace of mind from treatment with RAI	◯	◯	◯	◯	◯
**f.** Recommendation of other clinician	◯	◯	◯	◯	◯
**g.** Patient preference to **not** receive RAI	◯	◯	◯	◯	◯
**h.** Concern about **not** giving RAI	◯	◯	◯	◯	◯

16. In your opinion, is each of the following treatments for low-risk thyroid cancer overused, appropriately used, or underused in the United States?

	Overused	Appropriately used	Underused
**a.** Radioactive iodine	◯	◯	◯
**b.** Total thyroidectomy with central neck dissection	◯	◯	◯
**c.** Total thyroidectomy alone	◯	◯	◯
**d.** Thyroid lobectomy	◯	◯	◯
**e.** Active surveillance	◯	◯	◯

17. Finally, we have a few questions about you and your practice.

How many years have you practiced, since you finished your training? ________ years

© 2022 Dedhia PH et al. *JAMA Network Open.*

18. What is your age? ☐ years

19. Which of the following describes how you think of yourself?

- ◯ Female
- ◯ Male
- ◯ In another way: Please tell us: → ☐

20. Which of the following best describe your race or ethnicity? ***(Please check all that apply)***

- ◯ American Indian or Alaskan Native
- ◯ Asian
- ◯ Black or African American
- ◯ Hispanic or Latino
- ◯ Native Hawaiian or Other Pacific Islander
- ◯ White
- ◯ Other: Please tell us: → ☐

21. Which one of the following best describes the setting in which you practice?

- ◯ Academic tertiary hospital
- ◯ Academic-affiliated hospital
- ◯ Community hospital
- ◯ Private practice
- ◯ Other setting → Please tell us: ☐

22. In your practice, do you…

	Yes	No	Not applicable
**a.** …have access to a tumor board to discuss your patients?	◯	◯	◯
**b.** …personally perform thyroid ultrasounds on your patients?	◯	◯	◯
**c.** …ever refer your patients to a high-volume thyroid surgeon?	◯	◯	◯

23. Do you use the following clinical guidelines in your practice?

	Yes	No
**a.** American Thyroid Association 2015	◯	◯
**b.** American Association of Clinical Endocrinologists 2016	◯	◯
**c.** National Comprehensive Cancer Network 2017	◯	◯

d. Other guidelines? → Please tell us: ☐

24. What is the zip code where your practice is located? ☐ 5-digit zip code

Thank you for participating in this survey! Please return the questionnaire in the postage-paid envelope provided.

© 2022 Dedhia PH et al. *JAMA Network Open.*
